# Delivering Health Coaching in a Student‐Led Telehealth Clinic: Evaluating the Impact on Health‐Related Quality of Life

**DOI:** 10.1155/ijta/2228574

**Published:** 2026-06-09

**Authors:** Kristie Matthews, Terry Haines

**Affiliations:** ^1^ School of Primary and Allied Health Care, Monash University, Melbourne, Australia, monash.ac.za; ^2^ Monash Centre for Scholarship in Health Education, Monash University, Melbourne, Australia, monash.ac.za

**Keywords:** allied health, health coaching, quality of life, student-led clinic, telehealth

## Abstract

Health professional student–led clinics are commonly integrated into programmes of study to provide unique learning opportunities for students and provide improved access to care for underserved populations. Participant health outcomes from student‐led clinics are infrequently reported and are hard to compare with outcomes from other intervention approaches. This study aims to examine change in participant health–relate‐d quality of life following engagement in a telehealth mediated health coaching focussed student‐led clinic in Australia. Pre–post data using the EQ‐5D‐5L instrument were collected from 369 participants in the student‐led telehealth clinic between August 2020 and August 2024. Participant data were converted to a utility score relevant to an Australian population. The mean change in health utility score after participating in the clinic was 0.06 (95% CI 0.03–0.08, *p* < 0.001). Regression analyses showed that a significant positive change in mean utility score was observable in younger (18–35) participants (0.13, 95% CI 0.05–0.21, *p* = 0.001), females (0.06, 95% CI 0.02–0.09, *p* = 0.001) and participants with a lower baseline (< 0.6) utility score (0.14, 95% CI 0.07–0.20, *p* < 0.001). Significant change was observed across physical health, psychological and environmental concerns. As health coaching is known to be an important feature of preventative health strategies, this research is a promising indicator of how a student‐led telehealth clinic may be an effective contributor to the health care ecosystem.

## 1. Introduction

Student‐led clinics involve students enrolled in health professions courses directly providing health care, as volunteers or as part of a placement experience, under the guidance of a qualified health professional [1–3]. Student‐led clinics expand learning opportunities for students and have been reported to enhance the development of clinical skills, teamwork, personal accountability and person‐centred care [3, 4]. The focus of a student‐led clinic may be primarily educative, but it may also be established to align with a specific health or community need as a form of social enterprise [5]. Student‐led clinics provide real‐world experience for students in a structured and supported environment and can additionally improve access to care for underserved populations [3, 5, 6].

Investigations of patient or client outcomes from student‐led clinics are generally positive; however, the assessments and interventions employed for a given population are inconsistent, limiting comparison with other treatment approaches [6]. A recent review examining the cost‐effectiveness of student‐led clinics has highlighted the need for greater reporting on health outcomes in this area, particularly the outcome of health‐related quality of life, which is applicable across a broad range of intervention types [7]. Closer examination of these aspects within student‐led clinics would better allow changes in health outcomes to be compared between population and intervention approaches [8]. This would enable clinic providers and policy makers to see whether student‐led clinics are primarily for educational purposes or whether they can legitimately claim to form a part of the health care ecosystem.

An area of rapid development in health service delivery, accelerated by the COVID‐19 pandemic, is telehealth [9]. Using telehealth intentionally to underpin a student‐led clinic is unique. During the pandemic, several existing student‐led clinics transitioned to telehealth methods to sustain continuity of care for clients and learning opportunities for students [10–15]. Some telehealth‐specific student‐led clinics were also established during the pandemic to provide temporary outreach support to populations vulnerable to COVID‐19 [16–18] or to support preassessment of patients experiencing delays for specialist care [19]. The outcomes of these clinics have been reported primarily as enhancing access to care [10–12, 16–19] or student learning [13–15]. Health outcomes from telehealth‐based student‐led clinic models have not been reported in the literature.

The aim of this study was to investigate the impact on participant‐reported health‐related quality of life following engagement in a student‐led telehealth clinic operated by an Australian university and to examine factors that may moderate this effect.

## 2. Methods

This research used a prospective, quasi‐experimental, pre–post intervention (within‐subject) cohort study design. The reporting of this research has been guided by the STROBE checklist for cohort studies.

### 2.1. Participants and Setting

This study was conducted in Australia. Any person over the age of 18 years living in Australia who consented to engage in the student‐led telehealth clinic was eligible to participate as a ‘clinic participant’ in this study. Participants were not required to have any specific health condition to engage in the clinic. ‘Student participants’ in the clinic as a cohort of the research are not the subject of this paper.

### 2.2. Clinic Structure

The free‐of‐charge student‐led telehealth clinic was established by the Monash University School of Primary and Allied Health Care in August 2020. The student‐led telehealth clinic involved students from the disciplines of occupational therapy, paramedicine, physiotherapy, social work and podiatry. The intervention used video, telephone, or both to deliver telehealth consultations focused on health coaching conversations with clinic participants. The intervention was situated within a person‐centred, shared decision‐making framework [20], where the focus was on students and clinic participants working together to identify health and wellbeing concerns and goals for improvement that aligned with the needs of the participant.

Participation in the clinic contributed to student work–integrated learning activities within their course, and the duration of student involvement varied to align with course requirements. All students completed the same online prelearning modules and assessments prior to engaging with clinic participants. All prelearning activities were focussed towards supporting students to deliver health coaching conversations and interventions within the remit of a student‐led clinic, including knowing escalation pathways for any situation that presented beyond the students’ scope of practice.

Students were guided in their telehealth interactions with each participant by scripted prompts and data forms embedded within a purpose‐built customer relationship management database designed for the student‐led telehealth clinic. Telehealth interactions were initially stepwise (Table 1), with later flexibility for the student to apply an interaction type that aligned with the needs expressed by the clinic participant (i.e., to modify goals or to start supporting a new concern). Generally, it was intended that each participant–student pair had four to five telehealth interactions during a 4–6‐week period. However, this varied due to participant availability to engage and student scheduling in the clinic. Additionally, some participants chose to remain in the clinic for a longer period of time to address multiple health and wellbeing needs, engaging with successive students. This scenario was supported by templated documented handover using the ISBAR (Identity, Situation, Background, Assessment, Request) mnemonic [21] between students to maintain continuity of care. Participants could withdraw from the clinic at any time, either by choice or because the goals aligned to the selected problem had been achieved. On exit from the clinic, participants were invited to complete a phone‐based evaluation survey with the clinic administrator.

**Table 1:** Student‐led telehealth clinic interaction stages and purpose.

Telehealth interaction	Purpose
Screening	Baseline data of participant health and wellbeing status using several common assessment tools. Data collection included EQ‐5D‐5L. Screening was completed once, as the participant entered the programme.
Problem identification	Baseline understanding of current health and wellbeing concerns experienced by the participant. Intent was to select one problem to focus attention on initially. Subsequent problems may be selected later if necessary. Selected problems were described in the participant’s/student’s own words.
Previous strategies	Further understanding of any previous strategies attempted to address the problem. Informed strategies and goal setting in later interaction.
Strategies and goals	Discuss proposed strategies and goals to address the selected problem. Shared decision‐making between the participant and student regarding goals to try.
Goal review	Review to discuss if goals have been achieved and next steps. Optional continuation to discuss subsequent problems, strategies and goals as needed.

Each participant was allocated to the next available student within the clinic, unless the clinic participant made a specific request for a student discipline type at the time of consent. Students had an individual caseload of clinic participants that aligned with the needs of their course, and a same‐discipline student peer could be present during telehealth interactions as an observer to support later reflection and learning. Clinic supervisors were not present during telehealth interactions, but scheduled contact points were included in the structure for supervisors to facilitate case discussions and student decision‐making. Interprofessional learning [3] between students from different disciplines occurred opportunistically; however, student scheduling meant it was not feasible for this to be a predetermined activity.

### 2.3. Recruitment and Data Collection

This research utilised data collected during the period between August 2020 and August 2024. Ethics approval was granted by the Monash University Human Research Ethics Committee (Project Number 24722).

Clinic participants were recruited through a social media advertising campaign linked to an online expression‐of‐interest process. Respondents were then followed up by phone and read an explanatory statement by the clinic administrator and were provided with the opportunity to ask questions about the clinic. Consent to participate was recorded electronically in the customer relationship management database by the clinic administrator, along with basic demographic information (name, date of birth, and address). As prior health information was not available at the time of consent, clinic participants were allocated to the next available student in the database, and participants and students engaged in telehealth interactions as described in Table 1.

During the data collection period, 349 students were engaged in the clinic from the disciplines of paramedicine (*n* = 172), occupational therapy (*n* = 100), social work (*n* = 40), physiotherapy (*n* = 28) and podiatry (*n* = 9). The duration of engagement for each student varied from 3 weeks to 12 weeks, with a range of 1–4 days per week.

The primary outcome measure for this study was the EQ‐5D‐5L [22]. The EQ‐5D‐5L is a self‐rated health‐related quality of life survey that involves selection of health states across domains of mobility, self‐care, usual activities, pain/discomfort and anxiety/depression, along with completion of a visual analogue scale (VAS) of overall self‐rated health. The EQ‐5D‐5L has been used to measure health‐related quality of life across various populations and disease cohorts with good reliability and sensitivity [23].

Preintervention participant EQ‐5D‐5L data were collected by students during the telehealth screening interaction. Postintervention participant EQ‐5D‐5L data were collected by the clinic administrator during the exit survey approximately 2 weeks after the last interaction with a student. All participant responses were recorded in the customer relationship management database.

Demographic variables captured included participant gender, age, location of residence and discipline of the allocated student. Students also recorded as text the health and wellbeing problems identified by the participant that were focussed on in the clinic. All data were recorded in the customer relationship management database.

### 2.4. Data Analysis

Pre‐ and postintervention EQ‐5D‐5L responses were converted to a health utility score using an algorithm developed from the Australian population [24]. This algorithm generates a utility score where 1 indicates full health and 0 indicates death. Of the four models described (A–D), Model D was selected, as according to Norman et al. [24], it was most aligned with the monotonic nature of the EQ‐5D‐5L tool and most effectively reflected the impact on utility if any score was at Level 5 (i.e., the worst state of the five‐point EQ‐5D‐5L scale). The pre‐ and post‐VAS scores were maintained as a single integer between 0 and 100, as reported by participants.

Participant‐identified problems were coded using a deductive content analysis method [25] to align with the four domains of the WHOQOL‐BREF [26] (i.e., environment, physical health, psychological and social relationships). For example, the problems of ‘back pain’ or ‘fatigue’ would both be coded as ‘physical health’, and the problems of ‘anxiety’ or ‘stress’ would be coded as ‘psychological’. Coding was completed using NVivo14 (Lumivero) and transposed into Stata 15 (StataCorp LLC).

The effect of the telehealth clinic intervention on change in health utility and VAS scores was examined for the clinic participant cohort overall using univariate linear regression analyses. Data were clustered within participants, and robust variance estimates were employed to calculate 95% confidence intervals (CIs) and *p* values. This was to take into account the dependency of multiple observations within the same participant. We also examined a range of interaction effects to determine if the effect of the intervention differed across different subgroups. These included the clinic participant age group, the type of problem that participants presented with, gender, the discipline of the student participant they interacted with and the preintervention utility group. This latter variable was generated by clustering preutility scores into three groups representing different health states (i.e., > 0.8 as good health; 0.6–0.8 as moderate health; < 0.6 as poor health). Quantitative data analyses were conducted using Stata 15 (StataCorp LLC).

## 3. Results

A total of 879 clinic participants consented to participate in the student telehealth clinic between August 2020 and August 2024. Of these, 690 commenced telehealth interactions with a student, and the remaining 189 were waiting to start. Of the 690 who commenced, 655 completed the clinic, and 35 were still engaged with students at the time of data extraction, so they were excluded from comparisons of pre‐ and postmeasures. A total of 369 clinic participants (56%) agreed to complete the exit survey with the clinic administrator, whereas 286 declined to complete the survey or were unable to be contacted after they had completed the clinic. Both pre‐ and postintervention utility score data were available for 369 clinic participants, whose demographic data are presented in Table 2. Half of all participants came from Victoria despite the advertising campaign being nationwide, indicating that the algorithm promoting the study may have targeted more Victorians as being in the same location as the student‐led clinic. Eighty‐six participants (23%) resided in areas categorised as rural or remote locations according to the Modified Monash Model (Categories 3–7) [27].

**Table 2:** Demographics of people in the telehealth clinic intervention who completed both pre‐ and postassessments.

Item		*N* (%)
Age	Range 18–89 years (mean 53.2)	
	Not reported	17 (4%)

Gender	Male	52 (14%)
	Female	309 (84%)
	Nonbinary	0 (0%)
	Not reported	8 (2%)
	Total	369

Location of residence (state)	Australian Capital Territory	7 (2%)
	New South Wales	78 (21%)
	Northern Territory	5 (1%)
	Queensland	37 (10%)
	South Australia	18 (5%)
	Tasmania	4 (1%)
	Victoria	186 (50%)
	Western Australia	22 (6%)
	Not reported	12 (3%)

The mean period of time for participants in the student‐led clinic was 48 days (SD = 64). The type of problems participants worked on with the students in the clinic is presented in Table 3. A majority of people worked on a single problem during the clinic (84%); however, two people worked on five unique problems. Of problem types, the majority were physical health (56%), followed by psychological (29%), environment (11%) and social relationships (3%).

**Table 3:** Number of people who chose to work on multiple problems during the telehealth clinic intervention and the type of problems that were worked on.

Number of problems selected	Number of people	Type of problems			
		Environment	Physical health	Psychological	Social relationships
1	310	37	159	85	8
2	41	9	43	26	4
3	14	1	31	10	0
4	2	1	3	2	2
5	2	2	6	2	0
Total	369	50	242	125	14
		Total = 431			

The mean participant health utility score prior to starting the clinic was 0.67 (SD = 0.23), and the mean VAS score was 70.4 (SD = 16.2). After participating in the clinic, the mean participant health utility score was 0.73 (SD = 0.25), and the mean VAS score was 75.1 (SD = 16.3). The mean change in utility was 0.06 (95% CI 0.03–0.08, *p* < 0.001), which represents a standardised mean difference of 0.24 (95% CI 0.12–0.32). The mean change in VAS was 4.7 (95% CI 3.1–6.2, *p* < 0.001), which represents a standardised mean difference of 0.28 (95% CI 0.19–0.38).

The effect of the telehealth clinic intervention on change in health utility and VAS scores for each variable (age, gender, student discipline, baseline utility and type of problem) is presented in Tables 4, 5, 6, 7 and 8. Positive change was observed with all variables, although with a small‐to‐medium effect size. Significance (*p* < 0.05) in change to health utility and VAS scores was observed in people younger than 35 years and between 51 and 65 years (Table 4), females (Table 5), occupational therapy as student discipline (Table 6) and a baseline utility score of < 0.8 (Table 7). Environment, physical health and psychological problems all showed significance in change (Table 8).

**Table 4:** Effect of the telehealth clinic intervention on change in health utility and VAS scores by age in people who completed both pre‐ and postassessments.

Age (*n*)	Preutility: mean (SD)	Postutility: mean (SD)	Change in utility: mean difference (95% CI, *p* value)	Pre‐VAS: mean (SD)	Post‐VAS: mean (SD)	Change in VAS: mean difference (95% CI, *p* value)
18–35 (*n* = 47)	0.68 (0.20)	0.81 (0.17)	0.13 (0.05–0.21), *p* = 0.001	68.1 (15.2)	78.2 (11.5)	10.1 (4.6–15.7), *p* < 0.001
36–50 (*n* = 73)	0.68 (0.21)	0.74 (0.27)	0.06 (−0.1 to 0.14), *p* = 0.11	67.7 (15.5)	71.5 (16.9)	3.8 (−1.5 to 9.1), *p* = 0.15
51–65 (*n* = 123)	0.67 (0.20)	0.74 (0.23)	0.07 (0.01–0.12), *p* = 0.013	70.0 (15.7)	74.8 (15.9)	4.8 (0.8–8.8), *p* = 0.017
66–89 (*n* = 109)	0.67 (0.28)	0.69 (0.28)	0.01 (−0.05 to 0.09),*p* = 0.65	72.8 (17.5)	75.7 (17.8)	2.9 (−1.8 to 7.6), *p* = 0.22

*Note:* *n* = 17 did not choose to record their age.

**Table 5:** Effect of the telehealth clinic intervention on change in health utility and VAS scores by gender in people who completed both pre‐ and postassessments.

Gender (*n*)	Preutility: mean (SD)	Postutility: mean (SD)	Change in utility: mean difference (95% CI, *p* value)	Pre‐VAS: mean (SD)	Post‐VAS: mean (SD)	Change in VAS: mean difference (95% CI, *p* value)
Male (*n* = 52)	0.65 (0.31)	0.66 (0.29)	0.01 (−0.10 to 0.13), *p* = 0.81	64.1 (19.8)	71.3 (18.5)	7.2 (−0.1 to 14.7), *p* = 0.056
Female (*n* = 309)	0.68 (0.21)	0.74 (0.24)	0.06 (0.02–0.09), *p* = 0.001	71.4 (15.3)	75.5 (15.9)	4.1 (1.5–6.5), *p* = 0.001

*Note:* *n* = 8 did not choose to record their gender.

**Table 6:** Effect of the telehealth clinic intervention on change in health utility and VAS scores by discipline of the student in people who completed both pre‐ and postassessments.

Student discipline (*n*)	Preutility: mean (SD)	Postutility: mean (SD)	Change in utility: mean difference (95% CI, *p* value)	Pre‐VAS: mean (SD)	Post‐VAS: mean (SD)	Change in VAS: mean difference (95% CI, *p* value)
Occupational therapy (*n* = 256)	0.67 (0.23)	0.75 (0.25)	0.08 (0.03–0.12), *p* < 0.001	70.3 (15.5)	75.2 (16.0)	4.9 (2.1–7.6), *p* < 0.001
Paramedicine (*n* = 53)	0.68 (0.18)	0.68 (0.22)	0.00 (−0.08 to 0.07), *p* = 0.91	69.0 (16.8)	72.6 (17.7)	3.6 (−3.0 to 10.3), *p* = 0.28
Physiotherapy (*n* = 31)	0.71 (0.20)	0.74 (0.23)	0.03 (−0.08 to 0.13), *p* = 0.61	69.4 (19.4)	75 (16.1)	5.6 (−3.5 to 14.6), *p* = 0.22
Social work (*n* = 27)	0.64 (0.36)	0.68 (0.33)	0.04 (−0.15 to 0.23), *p* = 0.67	75.1 (16.3)	77.7 (16.0)	2.6 (−6.3 to 11.5), *p* = 0.56
Podiatry (*n* = 2)	0.88 (0.05)	0.95 (0.06)	0.07 (−0.17 to 0.31), *p* = 0.34	67.5 (38.8)	85.0 (7.0)	17.5 (−102 to 137), *p* = 0.59

**Table 7:** Effect of the telehealth clinic intervention on change in health utility and VAS scores by baseline utility score in people who completed both pre‐ and postassessments.

Baseline utility (*n*)	Preutility: mean (SD)	Postutility: mean (SD)	Change in utility: mean difference (95% CI, *p* value)	Pre‐VAS: mean (SD)	Post‐VAS: mean (SD)	Change in VAS: mean difference (95% CI, *p* value)
< 0.6 (*n* = 110)	0.39 (0.20)	0.53 (0.29)	0.14 (0.07–0.20), *p* < 0.001	57.7 (17.3)	63.3 (18.3)	5.6 (0.8–10.3), *p* = 0.022
0.6–0.8 (*n* = 144)	0.71 (0.05)	0.76 (0.18)	0.05 (0.02–0.08), *p* = 0.001	72.9 (11.9)	77.8 (12.8)	4.9 (2.0–7.8), *p* = 0.001
> 0.8 (*n* = 115)	0.90 (0.06)	0.90 (0.13)	0.00 (−0.03 to 0.01), *p* = 0.53	79.2 (11.9)	82.8 (11.0)	3.6 (0.6–6.6), *p* = 0.018

**Table 8:** Effect of the telehealth clinic intervention on change in health utility and VAS scores by type of problem in people who completed both pre‐ and postassessments.

Problem (*n*)	Preutility: mean (SD)	Postutility: mean (SD)	Change in utility: mean difference (95% CI, *p* value)	Pre‐VAS: mean (SD)	Post‐VAS: mean (SD)	Change in VAS: mean difference (95% CI, *p* value)
Environment (*n* = 50)	0.63 (0.23)	0.73 (0.22)	0.10 (0.00–0.18), *p* = 0.037	66.3 (17.6)	71.6 (18.4)	5.3 (−1.8 to 12.4), *p* = 0.14
Physical health (*n* = 242)	0.66 (0.24)	0.71 (0.26)	0.05 (0.00–0.09), *p* = 0.033	68.3 (17.5)	72.7 (17.1)	4.4 (1.3–7.4), *p* = 0.006
Psychological (*n* = 125)	0.64 (0.24)	0.72 (0.25)	0.08 (0.01–0.14), *p* = 0.011	67.5 (17.1)	75.1 (16.0)	7.6 (3.4–11.8), *p* < 0.001
Social relationships (*n* = 14)	0.69 (0.14)	0.81 (0.17)	0.12 (0.00–0.25), *p* = 0.051	74.0 (13.5)	76.6 (14.5)	2.6 (−8.3 to 13.4), *p* = 0.63

Significant interaction effects were observed when comparing a baseline utility score of < 0.6 with other groupings. Baseline utility Cohort 0.6–0.8 had a 1‐point difference in utility (*p* = 0.007), and Cohort > 0.8 had a 14‐point difference in utility (*p* < 0.001); however, significant interaction effects with VAS scores were not observed. We did not identify significant interaction effects when looking at change in utility across different problem types; however, a 3‐point difference was observed when comparing VAS scores between physical and psychological problem types (*p* = 0.04). Interaction effects when comparing utility and VAS scores for age, gender and student discipline types were not significant.

## 4. Discussion

This study has shown that a health coaching–focussed student‐led clinic delivered via telehealth to a general population can have an impact on participant self‐reported health‐related quality of life. It is understood that health coaching conversations delivered by health professionals can enhance physical and psychological wellbeing and support behaviour change in people with chronic health conditions [28]. Of relevance to this work, telephone‐based health coaching interventions have also demonstrated positive outcomes for this cohort [29]. In the context of our health coaching telehealth clinic delivered by students to a general population, it is notable that both physical and psychological problems showed significant improvement in utility and VAS scores, and environmental problems showed significant improvement in utility scores. Occupational therapy students were also shown to have the most significant influence on outcomes, arguably due to the holistic focus of discipline training across physical, psychological and social domains. It is also interesting to observe that the youngest‐age participant cohort (18–35)—and closest in age to many of the students—had the largest significant change in utility and VAS scores compared to other groups. Additionally, although existing chronic conditions were not a precursor to participating in the student‐led telehealth clinic, the impact of the clinic on health‐related quality of life in those presenting with worse quality of life at the start of the clinic (< 0.8 utility score) suggests this model may present a valid option for health care delivery. As health coaching conversations in chronic health contexts may project future cost‐savings to the health care system [30], it is important to consider how a student‐led clinic, particularly one using telehealth [31], may add value to health service provision.

Student‐led clinics commonly cater for a specific patient population with targeted outcomes, are in‐person and are time‐limited [6]. In contrast, the intention of this clinic was for students to lead participant‐directed health coaching conversations, using telehealth, for a period of time determined by the individual. Short‐term in‐person preventative health–focussed student‐led clinics for a general population are described in the literature, with outcomes presented as intentions for behaviour change [32–34]. In Australia, Stuhlmiller and Tolchard [35] described an indigenous community‐coproduced student‐led drop‐in general health clinic in a rural setting, with the authors indicating improvements in health literacy and health behaviours. In regional Australia, Walker et al. [36] delivered a preventative health interprofessional student‐led clinic to a population with physical chronic disease risk factors. The clinic was primarily delivered in‐person with participant attendance for a 4‐month period, with less than 5% of overall attendance via telehealth at the height of the pandemic [36]. From 146 participants, this clinic model demonstrated a mean change of 0.112 (*p* < 0.001) in preference‐adjusted quality of life outcomes (using the AQoL‐8D instrument) following participation [36]. A standardised mean difference in health‐related quality of life of 0.53 can be calculated from the Walker et al. study, which is in contrast to a standardised mean difference of 0.24 from the general population in the present study. However, when contrasting the Walker et al. population to the subgroup presenting with lower baseline health (< 0.6 utility score) in this study as a more akin population comparator, a standardised mean difference in health‐related quality of life of 0.48 (95% CI 0.24–0.68) is observed. Another comparison can be drawn with the findings of a meta‐analysis examining the impact of health coaching on quality of life in people with noncancer‐related pain, delivered by health professionals [37]. In this study, a pooled standardised mean difference of 0.19 (95% CI −0.14 to 0.53) is described. These contrasts indicate that our telehealth student‐led clinic approach may provide comparable impact on participant health and wellbeing to that of other student‐led clinics delivered in‐person, as well as to health coaching delivered by health professionals.

Improving the burden of chronic disease through preventative health care approaches is known to improve the cost burden on the health care system [8]; however, the potential impact of student‐led clinics and their contribution to health care are still an emerging area of research [38]. Mitchell et al. [7] have explicated the disparate nature of student‐led clinic outcome reporting in their systematic review, where benefits of the clinics in relation to patient health–related quality of life and quality‐adjusted life expectancy outcomes are uncommonly measured. Measuring such outcomes is vital if student‐led clinics are to be viewed as a useful contributor to improving population health [8]. However, despite the contribution that this study makes towards this goal, there remains an ongoing tension between supporting the provision of health care in a student‐led clinic without access to usual funding streams, in conjunction with supporting the provision of quality education experiences [39]. More research is needed to examine the health, education and economic impacts of student‐led clinics on all stakeholders.

There are several limitations to this study that should be acknowledged. In relation to the structure of the intervention, we are unable to observe the influence of time participating in the clinic (number of student interactions and total duration) on the outcomes measured. Additionally, the student discipline reflected in Table 6 reflects the final discipline contact that the participant experienced—we are unable to account for occasions of shared care where the participant may have been handed over between discipline types. Finally, we acknowledge the possibility of Type II errors in the outcomes in subpopulations where the sample size is small.

## 5. Conclusion

This study has shown that a student‐led telehealth clinic focussed on health coaching for participant‐directed health and wellbeing goals can positively impact participant‐reported health‐related quality of life. The telehealth clinic model was able to reach a geographically dispersed population and achieved outcomes comparable to those of an in‐person preventative health student–led clinic model and to health coaching delivered by health professionals. Although the economic delivery of the student‐led telehealth clinic has not been examined in this study, the opportunity to deliver impactful health care conversations without costly physical infrastructure and the burden of travel to a specific location by participants, students and academic supervisors is promising.

## Author Contributions

All authors contributed to the study conception and design. Material preparation and data collection were performed by Kristie Matthews. Data analyses were performed by the authors. The first draft of the manuscript was written by Kristie Matthews, and all authors reviewed and edited the manuscript.

## Funding

No funding was received for this manuscript. Open access publishing facilitated by Monash University, as part of the Wiley ‐ Monash University agreement via the Council of Australasian University Librarians.

## Disclosure

All authors read and approved the final manuscript.

## Ethics Statement

This study was approved by the Monash University Human Research Ethics Committee (Project Number 24722). All participants provided informed consent prior to engaging in this research.

## Conflicts of Interest

The authors declare no conflicts of interest.

## Data Availability

The data that support the findings of this study are available on request from the corresponding author. The data are not publicly available due to privacy or ethical restrictions.
